# Gold Fillings

**Published:** 1896-09

**Authors:** F. H. Skinner

**Affiliations:** Chicago, Ill.


ARTICLE VI.

### GOLD FILLINGS.

F. H. SKINNER, D. D. S., CHICAGO, ILL.

In proximate cavities separations are generally necessary. In bicuspids and molars the margins must be so cut that, after the filling is in, the line between gold and tooth structure will be self cleansing, or at least easily cleaned with the tooth-brush, and so that this line will in no place come in contact with the neighboring tooth. If the cavity does not extend far enough toward the gingiva to avoid this latter complication, cut sound tooth structure enough away so that the cervical margin of the filling will be easily accessible with a tooth-brush, or will be entirely covered by the gum.

Take a proximate cavity of an incisor; take a pellet of gold about the size of Rowan's three-fourths or smaller, if the size of cavity demands, and place over the linguo-gingival groove. With very finely serrated or smooth pluggers and small in diameter, with hand pressure gently press into the groove. This first piece is not condensed much. A second pellet of the same size is then annealed a little, and placed on the first pellet and condensed more. A part of this second pellet usually extends over the linguo gingival margin, or down toward it. This second pellet should, with light malleting or hand pressure, be fairly well condensed. In this way pellet after pellet is placed in the cavity, completely filling linguo-gingival and labio-gingival grooves and covering the gingival margin. By the time this is accomplished there is no danger of any rocking or loosening of gold already intact. Each pellet of gold will now be placed so that it extends slightly outside of the lingual border, and so that each piece extends more and more toward the cutting edge of the tooth, or what our friend Dr. Woolley calls, "crawling the gold." each pellet as it is placed should first be malleted against the wall of the cavity, with a small smooth or finely serrated point; then the part extending down over the lingual margin should be malleted against, or wrapped around the margin. For this, narrow, finely serrated foot pluggers, properly shaped to reach the required distance and angle, should be used, and all malleting should be done from the labial side of the tooth; for the lingual portion can never be properly built after the labial is completed, unless in irregularities. Therefore keep the gold well down and build the lingual wall complete and full as the filling progresses. Once the lingual wall fully covered and contoured, we have a very simple cavity which can be filled without difficulty, the only care being to thoroughly condense gold against the walls and have margins well covered. For durability of finish the last few pellets of gold should be some of the various brands of sheet gold, or, better still, heavy rooled gold, say number thirty, and should be thoroughly malleted. The next step will be to grind nearly down to the tooth with stones or disks or diamond points, the labial and lingual borders. Then with narrow strips, so as not to cut off the contour, finish down the gingival border, and then the border near the cutting edge.

Now, to make sure there are no soft places in the filling, steel burnishers should be used. This done, the filling may be brought down to its proper shape with coarse strips, and last a very fine one; and to gve it a final polish moose-hide cones or chamois skin disks and rouge is used.

As smoothness comes with high polish, too much pains cannot be taken in that direction. The filling, when finished, should be so contoured that nothing but the gold is in juxtaposition with the next tooth.—*Dental Review.*

---

ARTICLE VII.

### THE SUCCESSFUL DENTIST.

